# Revisiting cAMP signaling in the carotid body

**DOI:** 10.3389/fphys.2014.00406

**Published:** 2014-10-28

**Authors:** Ana R. Nunes, Andrew P. Holmes, Sílvia V. Conde, Estelle B. Gauda, Emília C. Monteiro

**Affiliations:** ^1^CEDOC, Chronic Diseases Research Center, NOVA Medical School/Faculdade de Ciências Médicas, Universidade Nova de LisboaLisboa, Portugal; ^2^School of Clinical and Experimental Medicine, University of BirminghamBirmingham, UK; ^3^Neonatology Research Laboratories, Department of Pediatrics, Johns Hopkins Medical Institutions, Johns Hopkins UniversityBaltimore, MD, USA

**Keywords:** cAMP signaling, carotid body, pharmacology, phosphodiesterase inhibitors, adenylyl cyclase, adenosine, dopamine, antipsychotics

## Abstract

Chronic carotid body (CB) activation is now recognized as being essential in the development of hypertension and promoting insulin resistance; thus, it is imperative to characterize the chemotransduction mechanisms of this organ in order to modulate its activity and improve patient outcomes. For several years, and although controversial, cyclic adenosine monophosphate (cAMP) was considered an important player in initiating the activation of the CB. However, its relevance was partially displaced in the 90s by the emerging role of the mitochondria and molecules such as AMP-activated protein kinase and O_2_-sensitive K^+^ channels. Neurotransmitters/neuromodulators binding to metabotropic receptors are essential to chemotransmission in the CB, and cAMP is central to this process. cAMP also contributes to raise intracellular Ca^2+^ levels, and is intimately related to the cellular energetic status (AMP/ATP ratio). Furthermore, cAMP signaling is a target of multiple current pharmacological agents used in clinical practice. This review (1) provides an outline on the classical view of the cAMP-signaling pathway in the CB that originally supported its role in the O_2_/CO_2_ sensing mechanism, (2) presents recent evidence on CB cAMP neuromodulation and (3) discusses how CB activity is affected by current clinical therapies that modify cAMP-signaling, namely dopaminergic drugs, caffeine (modulation of A_2A_/A_2B_ receptors) and roflumilast (PDE4 inhibitors). cAMP is key to any process that involves metabotropic receptors and the intracellular pathways involved in CB disease states are likely to involve this classical second messenger. Research examining the potential modification of cAMP levels and/or interactions with molecules associated with CB hyperactivity is currently in its beginning and this review will open doors for future explorations.

## Introduction

Adequate homeostatic regulation of arterial oxygen (P_a_O_2_), carbon dioxide (P_a_CO_2_), pH and blood glucose are important processes in physiology. Highly specialized chemosensory type I cells of the mammalian carotid bodies (CBs) sense acute changes in P_a_O_2_, P_a_CO_2_ and pH, and, upon stimulation, release neurotransmitters (NTs) that either inhibit or activate chemosensory fibers projecting into the central nervous system (CNS). The functional consequence of CB stimulation is the initiation of important cardiovascular, respiratory and metabolic reflexes. These reflexes include an increase in minute ventilation, a sympathetically mediated elevation in heart rate and peripheral vasoconstriction and an augmentation in adrenaline release from the adrenal medulla, with the latter leading to an increase in arterial blood glucose concentration.

Recently, interest in CB physiology has attracted considerable attention because of its emerging associations with chronic cardiovascular disease (McBryde et al., 2013). CB dysfunction and increases in chemoafferent discharge promote neurogenic hypertension in sleep disordered breathing (Prabhakar and Peng, 2004), chronic heart failure (Schultz et al., 2013) and essential hypertension (Abdala et al., 2012; McBryde et al., 2013). Moreover, the CB is a principal regulator in initiating insulin resistance in animal models of prediabetes and metabolic syndrome (Ribeiro et al., 2013). Therefore, the modulation of CB function may be necessary to prevent and treat some of these conditions. A good understanding on the modulation of the cellular processes occurring downstream of the CB transduction machinery, may not only promote drug development that modify CB chemodischarge to prevent or treat disease, but will also increase the awareness that CB chemodischarge can be an inadvertent side effect of drugs used to treat other diseases.

CB type I cells contain molecular sensors that, when activated, trigger transduction cascades that produce cellular depolarization, Ca^2+^ influx and NT and/or neuropeptide secretion. The list of characterized NTs/neuromodulators (NMs) and respective receptors in the CB has increased considerably over the last 20 years (Table 1). These NTs/NMs have the potential to activate metabotropic and ionotropic receptors located on type I cells (autoreceptors), on afferents of the carotid sinus nerve (CSN, post-synaptic receptors), or both, exerting either excitatory or inhibitory actions (Table 1). The activation of excitatory postsynaptic receptors is translated into an increase of CSN action potential frequency, and it is this signal that is conveyed to the CNS. Stimulation of excitatory autoreceptors induces an increase in [Ca^2+^]_i_ and subsequent further release of NTs/NMs.

**Table 1:**
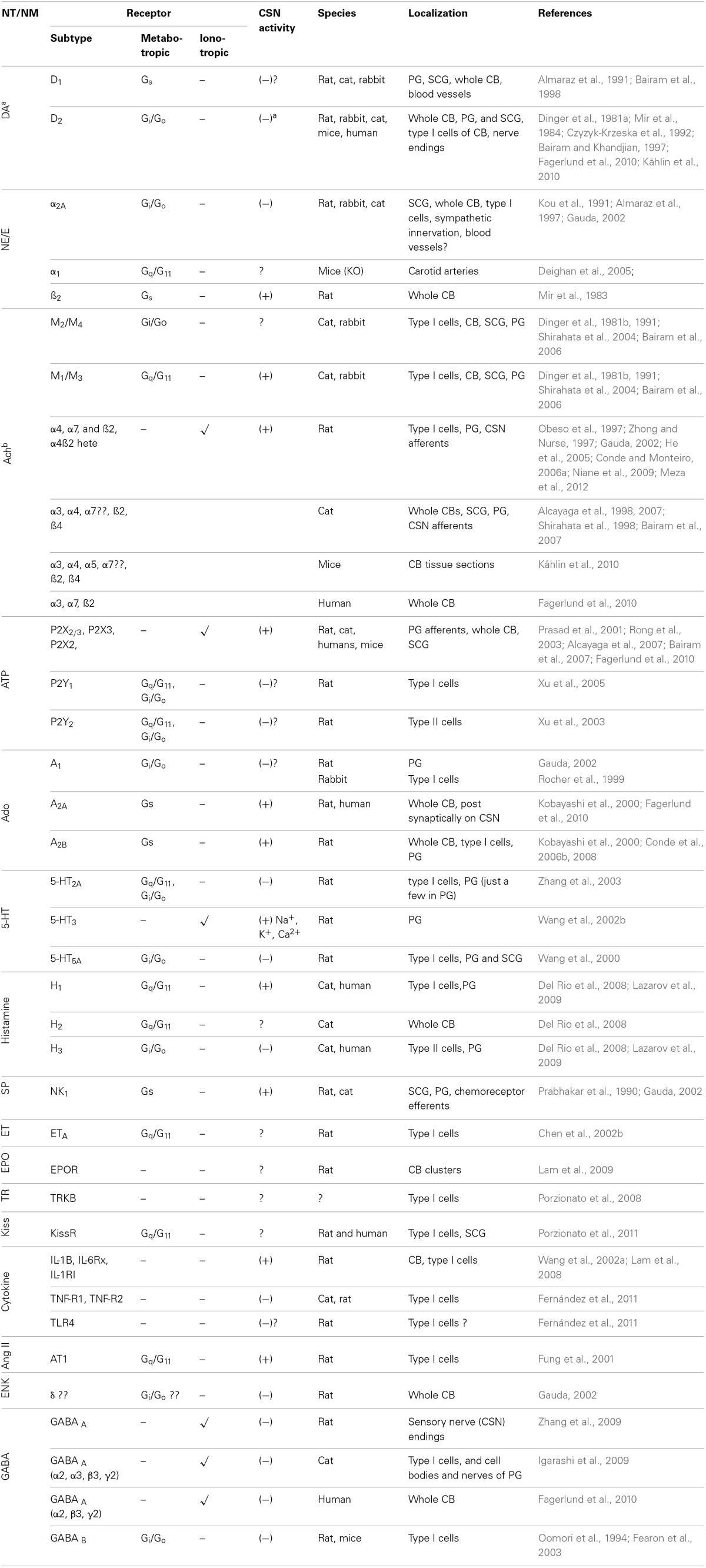
**Receptors in the carotid body**.

^a^Mainly inhibitory, but excitatory in rabbit (Iturriaga et al., 2009). ^b^Although less characterized in rat, the nAchRα3,4,5,7, and ß2,4 are present in type I cell, α7 in the CNS afferents and α3,4,7, and ß2,4 in PG; the mAchR M1 and M2 are in type I cells, M1 in CSN afferents and M1 and M2 in PG neurons of cat and rabbit (for a revision, Shirahata et al., 2007). ?, suggested, but no direct evidences/not known; NT/NM, neurotransmitters/neuromodulators; DA, dopamine; NE/E, norepiniphrine/epiniphrine; NE, norepiniphrine; Ach, acetylcholine; ATP, adenosine triphosphate; Ado, adenosine; 5-HT, serotonine; GABA, gamma-aminobutyric acid; ENK, enkephalins; SP, substancia P; ET, endothelins; TR, trophin; AngII, Angiotensin; AC, adenylyl cyclase; +, excitatory; −, inhibitory; CB, carotid body; SCG, superior cervical ganglion; PG, petrosal ganglion.

Retrograde communication between petrosal ganglion (PG) neurons and CB type I and type II cells is another source of NTs/NMs release in the CB. PG neurons present catecholaminergic traits (Katz et al., 1983; Katz and Black, 1986) and catecholamines (CAs) are released from cultured PG neurons upon stimulation (Iturriaga et al., 2003). Moreover, nitridergic autonomic neurons located in the glossopharyngeal and carotid nerve may also modulate the CB function (Campanucci et al., 2012). The pannexin-1 channel opening have been recently shown to be important in reciprocal cross-talk pathways between type I and type II cells, particularly in purinergic transmission (ATP and Ado) (Nurse, 2014).

The specific NT profile, receptor expression and cellular effects changes with early postnatal development, and in some cases exhibits interspecies variability [e.g., dopamine (DA) exerts inhibitory effects on the CB in most species, except rabbit, (Iturriaga et al., 2009)].

Despite the numerous different NTs/NMs released from the type I cell, even under basal conditions, a convergence upon a common signaling pathway could confer the overall CB excitability and establish its sensitivity to physiological stimuli. Cyclic adenosine monophosphate (cAMP) is a common downstream signaling molecule of numerous receptors expressed in the type I cells, and is coupled to cellular energetic status (AMP/ATP ratio). This article therefore aims to summarize how changes in CB cAMP levels in physiology, pathology and following pharmacological intervention may be central to alterations in type I cell excitability leading to chemoafferent discharge and cardiorespiratory and metabolic reflex responses.

## Classical understanding of cAMP-signaling pathway in the carotid body

An involvement of cAMP in the CB chemotransduction (late 70s-early 80s) was originally prompted by the identification of secreted NTs and their receptor-mediated effects on CB chemoreceptor responses (Table 1). These secreted NTs included DA (Gonzalez and Fidone, 1977), acetylcholine (ACh) (Eyzaguirre et al., 1965), noradrenaline (NA), substance P, serotonin and prostaglandin E_2_ (Pérez-García et al., 1993 for early references), and adenosine (Ado) (McQueen and Ribeiro, 1981; Monteiro and Ribeiro, 1987; Conde and Monteiro, 2004), acting through specific G-protein coupled receptors (Table 1). For those NTs that confer an excitatory response, cAMP levels were increased, while for those that confer an inhibitory response cAMP levels were decreased.

Fitzgerald and co-workers, were the first to identify cAMP in the CB cat homogenates (Fitzgerald et al., 1977). Subsequent studies showed that injection of isoprenaline increased cAMP accumulation in the rat CB (Mir et al., 1983) and elevated chemoafferent discharge frequency in cat and rabbit models via stimulation of beta-adrenoreceptors (Folgering et al., 1982). Moreover, administration of dibutyryl cyclic AMP (db-cAMP, cAMP analog) was found to mimic the excitatory effect of adenosine on chemosensory discharge (McQueen, 1983).

Following these findings, a new wealth of evidence emerged supporting a role for the cAMP in CB chemotransduction and/or chemotransmission (Wang et al., 1989, 1991a; Fidone et al., 1990; Pérez-García et al., 1990; Cachero et al., 1996; Summers et al., 2002). Multiple investigations reported rises in CB cAMP accumulation following hypoxia exposure (Fidone et al., 1990; Pérez-García et al., 1990), an effect that appeared to be specific for chemoreceptor tissue (Wang et al., 1989) and was dependent on NT release (Pérez-García et al., 1990). Activation of adenylyl cyclases (AC), by forskolin (FSK), potentiated CB CA secretion and CSN discharge frequency over a range of O_2_ tensions from 30 to 0%, in the intact rabbit CB preparation (Almaraz et al., 1991; Wang et al., 1991a). In addition, FSK and db-cAMP both inhibited the type I cell O_2_-sensitive K^+^ current, emphasizing similarities between excitatory cAMP and hypoxic signaling cascades (López-López et al., 1993). Hypercapnia exposure also elevated cAMP content (Pérez-García et al., 1990) and FSK augmented the hypercapnic CA release (Pérez-García et al., 1991). In isolated rabbit CB type I cells, cAMP analogs potentiated inward Ca^2+^ current in a manner that was comparable with hypercapnia (Summers et al., 2002). Despite these data, it was not universally accepted that endogenous cAMP was physiologically relevant in the CB.

Delpiano et al. reported, using an *in vitro* preparation of the cat CB, that anoxia exposure induced only small increases in cAMP levels (Delpiano and Acker, 1991). Furthermore, severe whole body hypoxia exposure caused both increases and decreases in CB cAMP accumulation (Delpiano and Acker, 1991), and short periods of hypoxia (2.5–5 min) failed to alter the cAMP levels in rat CB (Mir et al., 1983). K^+^ and Ca^2+^ currents, both important in hypoxic chemotransduction, were shown to be insensitive to an array of cAMP analogs in the rat CB type I cells (Hatton and Peers, 1996); inwardly rectifying Cl^−^ current is directly activated by cAMP (Carpenter and Peers, 1997). The inter-experiment variability, differences in species and age, in CB dissection methods, O_2_ and CO_2_ stimulus intensity, duration of incubation periods, CB preparations (*in vitro*, *in vivo*, whole CB vs. isolated cells or carotid sinus nerve (CSN) preparations) and cAMP detection methods (radioimmunoassay, enzyme-immunoassay, protein binding saturation assays) have all been credited for the discrepancies reported in the literature regarding the relevance of cAMP signaling in CB function (Table 2).

**Table 2:** **Effects of different work conditions on cAMP levels in the carotid body**.

**[cAMP]**	**Species**	**Anesthesia**	**Preparation**	**Basal conditions**				**Stimulus**				**Technique**	**Units**	**References**
				**O_2_ (%)**	**CO_2_ (%)**	**Time (min)**	**Inc.media**	**O_2_ (%)**	**CO_2_ (%)**	**Time (min)**	**Inc. media**			
=^a^	Cat 12 month	Na-pentob.	Whole CB, *In vivo, In vitro*	R.A.	R.A.	n/a	n/a	5	n/a	3/10/20	n/a	RIA	pmol/CB	Delpiano et al., 1984
				30	3	60/120	Locke's	0	3	2	Locke's + [IBMX] 0.8 mM			
↑	Cat 12month	Na-pentob.	Whole CB, *In vitro*	35	4	180	Locke's modified	3	4	2	Locke's modified + [IBMX] 0.8 mM	RIA	pmol/CB	Delpiano and Acker, 1991
↑	Rabbit adult	Na-pentob.	Whole CB, *In vitro*	100	0	30	Tyrodes's	5	0	10	Tyrodes's	RIA	pmol/mg tissue	Wang et al., 1989
↑	Rat adult	Na-pentob.	CB slices, *In vitro*	100	0	30	Locke's + [theophyline] 10 mM	4	0	10	Locke's + [theophylline] 10 mM	Immune-reactivity	% positive cells	Wang et al., 1991b
↑	Rabbit adult	Na-pentob.	Whole CB, *In vitro*	100/95	0/5	30	Tyrodes's	0/5/7/10	5/20	10	Tyrodes's + [FSK] 0.01 mM, [IBMX] 0.5 mM and [Ca^2+^] 2 mM	RIA	pmol/mg tissue	Pérez-García et al., 1990
↑	Rabbit adult	CO_2_	Whole CB, *In vitro*	21	5	20	HCO^−^_3_ enriched- medium	21	10	5	HCO^−^_3_ enriched- medium	EIA	pmol/μg protein	Summers et al., 2002
↑	Rabbit adult	Na-pentob.	Whole CB, *In vitro*	20	5	30	Tyrodes's modified + [HCO^−^_3_] 24 mM	7	5	10	Tyrodes's modified + [HCO^−^_3_ 24 mM + [IBMX] 0.5 mM	RIA	pmol/mg tissue	Cachero et al., 1996
↑	Rabbit adult	Na-pentob.	Whole CB, *In vitro*	100	0	30	Tyrode's modified	5	0	10	Tyrode's modified	RIA	pmol/mg tissue	Chen et al., 1997
=	Rat adult	Urethane	Whole CB, *In vivo*	R.A	R.A.	n/a	n/a	5	0	2/5	n/a	Protein binding	pmol/CB	Mir et al., 1983
=	Rat (3, 12, 24 months)	Na-pentob.	Whole CB, *In vitro*	95	5	15	Tyrodes's modified	95/20/10/5	5	30	Tyrodes's modified + [IBMX] 0.5 mM	EIA	pmol/mg tissue	Monteiro et al., 2011
=	Rat adult	Na-pentob.	Whole CB, *In vitro*	20/95	5	15	Tyrodes's modified	5	5	30	Tyrodes's modified + [IBMX] 0.5 mM	EIA	pmol/mg tissue	Nunes et al., 2010

a Small increases in cAMP levels were observed in hypoxia only in the absence of IBMX. Inc., incubation; pentob., pentobarbital; R.A., room air; n/a, not applicable; RIA, radioimmunoassay; Locke's (in mM): NaCl 128, KCL 5.6, CaCl_2_ 122.1, D-glucose 5.5, NaHCO_3_ 10 and Hepes 7; Tyrode's (in mM):NaCl 112, KCl 4.7, CaCl_2_ 2.2, MgCl_2_ 1.1, Na-glutamate 42, Hepes 5, glucose 5.6, pH 7.4; Tyrode's modified solution (in mM):NaCl 140, KCl 5, CaCl_2_ 2, MgCl_2_ 1.1, Hepes 10, glucose 5.5, pH 7.42; HCO^−^_3_ medium (in mM): NaCl 117, KCl 4.5, CaCl_2_ 2.5, MgCl_2_ 1, sucrose 10, glucose 11, HCO^−^_3_ 23, pH 7.42; CB, carotid body; EIA, Enzyme Immuno Assay; IBMX, Isobutyl-1-methylxanthine.

Thus, there was still a requirement to further characterize and better understand the physiological significance of cAMP in the CB. To consider cAMP signaling as a physiological modulator of the chemoreceptor activity, disruption of cAMP generation, metabolism, or its intracellular effectors would need to be synonymous with functional modification of basal CB activity and/or its responses to hypoxia/hypercapnia. [cAMP]_i_ is tightly regulated by AC, by enzymes involved in its degradation (phosphodiesterases; PDE), and by the fluctuating activity of downstream effectors (Kamenetsky et al., 2006). The AC activities are highly integrated and determined by receptor-mediated changes in G-stimulatory (G_s_) and G-inhibitory (G_i_) proteins as well as by CO_2_ and HCO^−^_3_ (depending on specific AC isoforms- see below). [cAMP]_i_ can also be modified by direct diffusion from one cell to another through gap junctions (Bevans and Harris, 1999) or through transport to the extracellular milieu where it produces regulatory functions in multiple tissues (for a review Bankir et al., 2002; Hofer and Lefkimmiatis, 2007). Downstream effectors classically include protein kinase A (PKA) (Taylor et al., 1990), cyclic nucleic gated ion channels (Craven and Zagotta, 2006) and exchange proteins activated by cAMP (EPACs) (De Rooij et al., 1998).

Recently, better research tools have become available to more accurately detect intracellular cAMP and its regulation, thereby allowing us re-examine the enzymatic regulation of cAMP within the CB and its intracellular targets, during normoxia, hypoxia and hypercapnia conditions.

## Novel findings characterizing the enzymatic regulation of cAMP accumulation in the carotid body

Over the last decade, the enzymes involved in the cAMP-pathway signaling in the CB have been identified, and their activity modulated by natural stimuli. Novel findings have been recently reported as to how O_2_/CO_2_ exposure affects the CB cAMP-signaling.

### The role of adenylyl cyclases in the carotid body activity

The AC are enzymes that catalyze the synthesis of cAMP through the cyclization of ATP. There are two main classes: the classic NT-sensitive transmembrane (tmAC) and the more recently described soluble adenylyl cyclase (sAC) (for a review see Kamenetsky et al., 2006). The activity of the former is primarily influenced by extracellular signals (e.g., NTs, hormones, pharmacological agents) and is further subclassified in terms of G-protein associations, Ca^2+^ related signaling pathways (Halls and Cooper, 2011) and more recently by CO_2_ interactions (Townsend et al., 2009; Cook et al., 2012). sAC is regulated directly by HCO^−^_3_ and Ca^2+^, in a pH independent manner, as being shown primarily in testis and further extended to other organs as described below (Chen et al., 2000; Jaiswal and Conti, 2003).

The presence of the different AC mRNA transcripts was only reported recently; intact rat CBs (16-17 postnatal days) express tmAC1, tmAC2, tmAC3, tmAC4, tmAC6 and tmAC9, with tmAC1, tmAC4 and tmAC6 exhibiting the highest fold expression level (Nunes et al., 2013). sAC mRNA has also been identified, and is expressed at greater levels in the CB than in non-chemosensitive neuronal tissues (Nunes et al., 2009). The studies on AC mRNA were performed in whole CB (containing type I and type II cells, vessels, nerve endings, etc.). Whether there is a specific and clearly distinguished physiological function for each AC isoform, in the CB is unknown. Many agents that target cAMP signaling pathways are also likely to non-selectively act on the respective G-protein coupled receptors or PDE, thus making individual AC targeting challenging. However, in other tissues, individual AC isoforms do demonstrate unique functionality and this has led to increased interest in identifying specific AC isoforms as potential drug targets (Pierre et al., 2009). Genetic association studies have been valuable in unraveling the importance of specific AC in physiology and disease. For instance, a polymorphism of AC6 have been associated with alterations in blood pressure and heart rate regulation in humans (Hodges et al., 2010). Point mutations of the AC3 gene are also associated with decreased insulin release in animal models of type 2 diabetes (Abdel-Halim et al., 1998). Correlating specific mutations in tmAC genes with CB dysfunction and hypertension across patient populations may help refine CB disease related research. This data is currently unavailable but could be invaluable given the emerging relevance of the CB in cardiovascular system pathology.

Although ATP binding to ionotropic receptors likely mediates excitatory chemodischarge to hypoxia, DA and Ado are two key participants in modifying type I cell and/or post-synaptic cAMP via their modification of tmAC activity. Hypoxia-induced raises in type I cell [Ca^2+^] and ^3^H-DA neurosecretion are depressed in the presence of specific D_2_ receptor agonists (Benot and López-Barneo, 1990; Carroll et al., 2005; Conde et al., 2008), an effect that is associated with a reduction in CB cAMP content in both conditions. Deficiency of D_2_ receptors in adult mice blunts type I cell neurosecretion, but not CSN responses to hypoxia, possibly consistent with opposing pre-synaptic and post-synaptic neuromodulation (Prieto-Lloret et al., 2007). Systemic inhibition of Ado receptors decreases, but does not abolish, the CB mediated acute phase of the hypoxic ventilatory response (Lee et al., 2005). Using *in vitro* CB preparations, Conde et al. reported that blocking Ado receptors depresses hypoxic induced CA release and chemoafferent activity, an effect that is greater in milder rather than severe hypoxic conditions (Conde et al., 2006b, 2012a). D_2_ receptors are negatively coupled to AC while Ado A_2B_ are positively coupled to AC. Blockage of Ado A_2B_ receptors counteract the decrease in cAMP elicited by D_2_ receptor activation suggesting an A_2B_ and D_2_ autoreceptor interaction accounting for overall [cAMP]_i_ in the type I cell (Conde et al., 2008). In acutely dissociated type I cells, Ado A_2A_ receptor inhibition abolishes the [Ca^2+^]_i_ elevations evoked by Ado (Xu et al., 2006). Since both A_2A_ and A_2B_ receptors exert their actions through excitation of tmACs (reviewed in Ribeiro and Sebastião, 2010), it is the increase in [cAMP]_i_, that is most likely to account for its overall chemostimulatory function. Accordingly, directly inhibiting tmACs with SQ22536, does indeed depress hypoxic induced CA-secretion (Rocher et al., 2009).

These findings do not, however, confine CB cAMP content to the regulation of DA and Ado. Essentially any NT/receptor system that is coupled to tmAC will alter cAMP levels in the CB, including histamine/H_1_ and H_3_ receptors (Del Rio et al., 2008, 2009; Thompson et al., 2010), adrenaline/β-adrenergic receptors (Mir et al., 1983; Hauton et al., 2013), pituitary adenylate cyclase-activating protein (PACAP)/PAC_1_ receptor (Xu et al., 2007; Roy et al., 2013), among others (also see Table 1).

sAC activity has been described in numerous tissues where changes in HCO^−^_3_/CO_2_ are essential to their function. For instance in the testis, where sAC is highly expressed, sAC mediates sperm maturation and acquisition of motility (Buck et al., 1999; Hess et al., 2005). In the kidneys it regulates recycling of V-ATPse (Pastor-Soler et al., 2003), in airway epithelial cells sAC regulates the ciliary beat frequency (Schmid et al., 2007), and in corneal endothelium it plays a role in the activation of the cystic fibrosis transmembrane conductance regulator (Sun et al., 2004). sAC mRNA has now been identified in the whole CB, and although the sAC mRNA cellular localization has not been demonstrated, it is expressed at greater level in the intact organ than in other non-chemosensitive neuronal tissues (Nunes et al., 2009, 2013).

The physiological role of sAC in CO_2_ sensing was only recently studied in the CB chemoreceptors and its function appears somewhat equivocal. Contrary to those that reported rises in cAMP content, and PKA dependent Ca^2+^ current during isohydric hypercapnia (Pérez-García et al., 1990; Summers et al., 2002), observations from our laboratory indicate that increasing the HCO^−^_3_/CO_2_ ratio from 24mM /5% (normocapnia) to 44mM/10% (isohydric hypercapnia) does not alter cAMP content, PKA activity or CSN discharge frequency and, under these conditions, these assays were insensitive to the sAC inhibitor KH7 (Nunes et al., 2013). We did however show that KH7 decreased cAMP content under basal conditions and we speculated that sAC contributes more to normocapnic rather than hypercapnic [cAMP]_i_, at least in the rat CB. Examining the extent of sAC HCO^−^_3_ saturation in normocapnia/normoxia and whether this alters type I cell [Ca^2+^]_i_, chemoafferent frequency or responses to hypoxia/acidosis may thus be an important area for future investigation.

### The role of phosphodiesterases in the carotid body activity

PDE catalyze the hydrolysis of the 3-cAMP phosphate bonds of adenosine 3,5—cyclic monophosphate to AMP. According to the pharmacological principle that the regulation of the second messenger degradation can often make a more rapid and larger percentage change in concentration than comparable regulation of the rates of synthesis, PDEs are important modulators of cAMP levels, preventing uncontrolled diffusion of cAMP through the cell and consequently contributing to the formation of localized pools or gradients of cAMP within the cell (Lugnier, 2006). There are 11 known distinct PDE isoforms, each displaying unique substrate affinity and variable adjustment to endogenous co factors and pharmacological inhibitors (Bender and Beavo, 2006).

Uncharacterized PDE was first identified in the CB in 1977 by Hanbauer and Lovenberg, and these studies provided the first evidence for an O_2_ dependent cAMP affinity (Hanbauer and Lovenberg, 1977). From then on, studies aiming to indirectly assay CB AC activity and manipulate cAMP levels in responses to different oxygen concentrations have been performed in the presence of the xanthine 3-isobutyl-1-methylxantine (IBMX), a non-selective PDE inhibitor (*k*_*i*_ = 1–10 mM, Dousa, 1999 and *IC*_50_ = 2–50 mM, Bender and Beavo, 2006) with potential to block Ado receptors (*k*_*i*_ = 7.28 mM, Daly et al., 1991). A particular limitation of IBMX is its inability to inhibit PDE7 and PDE8 (Lugnier, 2006).

The PDE4 isoform was recently proposed as a major regulator of cAMP-hydrolyzing activity in the rat CB (Nunes et al., 2010). PDE4 comprises four subtypes (PDE4A, PDE4B, PDE4C, and PDE4D) with at least 35 known splice variants (Bender and Beavo, 2006). Selective pharmacological inhibition of PDE4 increases CB cAMP content in normoxia and causes even greater rises during hypoxia. These increases are however considerably lower than those observed in the presence of IBMX, suggesting a physiological role of additional isoforms (Nunes et al., 2010). However, at the time of this review, no functional data relating PDE4 activity with CB responses to hypoxia or hypercapnia has been published; functional studies are necessary to further strengthen the position of PDE4 in the CB. Intriguingly, given that PDE4 activity increases with chronic hypoxia in O_2_-sensitive pulmonary arteries and blood (Maclean et al., 1997; Spoto et al., 1998; Millen et al., 2006), a compensatory role for PDE4 to counter CB hyperactivity, although speculative, is plausible. That said, the consequences of chronic hypoxia or chronic intermittent hypoxia exposure on PDE activity in the CB remains to be explored.

### The role of cAMP effectors in the carotid body activity: protein kinase A, exchange protein activated by cAMP and cyclic nucleotide gated channels

PKA is the classical downstream effector of cAMP. It is a holo-tetrameric serine/theonine kinase composed of two regulatory and two catalytic subunits. Four cAMP molecules bind to the regulatory subunits, each with two cAMP binding sites. The cAMP binding promotes the dissociation of the catalytic subunits that bind ATP to become catalytically active and phosphorylate serine and threonine residues in intracellular targets such as A-kinase anchoring proteins (AKAPS) and ion channels. AKAPs tether PKA to particular cellular organelles and to the plasma membrane confining the PKA signaling to a small pool within the cells (Beene and Scott, 2007). In the nucleus, PKA can phosphorylate transcription factors, such as cAMP response element binding protein (CREB), and thus regulate gene expression.

A physiological role for PKA in CB chemotransmission is at present controversial. In dissociated rabbit type I cells, PKA inhibition by PKA inhibitor (PKAi) diminishes the rise in L-type Ca^2+^ current in response to isohydric hypercapnia (Summers et al., 2002). However, we reported that PKA activation status, as measured by Fluorescent Resonance Energy Transfer (FRET) based reporters, is unaltered during isohydric hypercapnia in isolated rat type I cells (Nunes et al., 2013). Multiple blockers of PKA have no effect on hypoxic CA-secretion in the intact rat CB preparation (Rocher et al., 2009). In contrast, acute rises in type I cell [Ca^2+^]_i_ evoked by Ado and PACAP are essentially abolished by PKA inhibition with H89 (10 μM) (Xu et al., 2006, 2007). Sustained plateau CSN activity mediated by PACAP is inhibited by only 41% in the presence of H89, in a preparation including the carotid bifurcation- CB-CSN-superior cervical ganglion (Roy et al., 2013). Furthermore, type I cell activation by methylcholine is sensitive to tmAC inhibition but not H89 inhibition, which suggests that cAMP signaling cascades in the CB are independent of PKA activation (Thompson and Wyatt, 2011). Whether these multiple discrepancies reflect fundamental species differences, different preparations (whole CB vs. cultures or type I cells) or other unidentified experimental factors are unclear, but precaution must be taken when using H89 due its reported non-specific inhibitory effect (Lochner and Moolman, 2006). In cellular preparations, it is likely that transmitters released from type I cells are lost to the superfusate and so their potential excitatory or inhibitory autoregulation of the type I cell chemosensitivity to hypoxia or hypercapnia is not apparent. Additionally, the contribution of retrograde communication (PG neurons to CB cells) should be also taken in account (Katz et al., 1983; Katz and Black, 1986; Iturriaga et al., 2003) as well as the new concept of tripartite sensory synapse between type I, type II and PG neurons (Nurse, 2014). Also, we now know that the contribution of the NTs to the hypoxic chemosensitivity in the CB depends on hypoxic intensity meaning that different hypoxic intensities will evoke the release of different NTs (Conde et al., 2012a) and therefore the differences observed in PKA activation can reflect distinct hypoxic intensities/mediators involved.

Different effects of PDE4 inhibitors on cAMP accumulation induced by hypoxia (Nunes et al., 2010), could suggest a different degree of PDE phosphorylation induced by differences in PKA activity mediated by hypoxia (Bender and Beavo, 2006).

Complex cAMP driven mechanisms through PKA and extracellular signal regulated kinase (ERK) mediated phosphorylation can modify PDE4 specific isoforms activity and subsequently alter the sensitivity to selective inhibitors (Bender and Beavo, 2006). Thus, determining whether acute hypoxia causes PDE4 activation by PKA or ERK mediated phosphorylation would be of interest.

EPAC is a guanine nucleotide exchange factor (GEF) for the RasGTPase homologs, Rap1 and Rap2. EPAC is composed of two regions: an N-terminal regulatory region containing a cAMP-binding site and a C-terminal catalytic region, with GEF activity (De Rooij et al., 1998). In the inactive conformation, EPAC is folded and the regulatory domain functions as an auto-inhibitory domain. cAMP binding unfolds the protein, allowing Rap to bind (for a review Gloerich and Bos, 2010). Rap GTPases cycle between an inactive GDP-bound and an active GTP-bound state, with GEFs mediating the exchange of GDP for GTP. GTPase-activating proteins then convert Rap to the inactive form. The activated Rap-GTP activates a variety of different mechanisms in the cell: promotes integrin-mediated cell adhesion, gap junction formation and ERK_1/2_ MAPK-mediated protein phosphorylation, stimulates phospholipase C-ε which hydrolyzes PIP2 to generate diacylglycerol, and the Ca^2+^ mobilizing second messenger IP_3_ (for a review Holz et al., 2006).

Rocher and co-workers initially proposed a physiological role for EPAC in the CB by examining the effects of the EPAC activator (8-pCPT-2′-O-Me-cAMP) and inhibitor (brefeldin) on the release of CAs (Rocher et al., 2009). Specifically, 8-pCPT-2′-O-Me-cAMP reversed the action of SQ22536 and brefeldin inhibited CA-secretion during hypoxia by approximately 50%. These authors suggested that the effectors of EPAC were likely to be the exocytotic machinery and K^+^ channels (Rocher et al., 2009). More recently, this group has identified the expression of both EPAC1 and EPAC2 in the rat CB (Ramirez et al., 2012). In addition, EPAC activation by cAMP is proposed to cause downstream stimulation of the IP_3_ receptor in the endoplasmic reticulum (Thompson and Wyatt, 2011) along with activation of PKC (Roy et al., 2013). Thus, crosstalk between the G_s_/G_i_ (cAMP-related) and G_q_ signaling pathways within the type I cell likely occurs. Better characterization of this interaction could be particularly insightful given the known upregulation of G_q_ signaling associated with CB dysfunction in sleep disorder breathing (Peng et al., 2006) and CHF (Li et al., 2006).

cAMP can directly bind to cyclic nucleotide-gated (CNGC) and hyperpolarization-activated cyclic nucleotide-modulated (HCNC) ion channels. These channels belong to a superfamily of voltage-gated cation channels, and thus the binding of cAMP to these channels is translated into changes in membrane potential and influx of Ca^2+^ and Na^+^. By conducting Ca^2+^, they can stimulate Calmodulin (CaM) and CaM-dependent kinases and, in turn, modulate cAMP production by regulating activity of AC and PDE. Since CNGC and HCNC are also permeable to Na^+^ and K^+^, they can also alter the membrane potential in electrically active cells. The presence of these channels in the rat CB has been suggested by the work of Stea and co-workers (Stea et al., 1995); however, others have reported that cAMP analogs do not affect Ca^2+^ currents in type I cells (López-López et al., 1993). HCNC ion channels have not been characterized in the CB.

cAMP can be released from a variety of cell types and tissues (for a review see Bankir et al., 2002). Transport of cAMP moves against a concentration gradient, that is temperature dependent, unidirectional and requires energy (Rindler et al., 1978). One of the proposed functions of the extracellular cAMP is to regulate extracellular Ado levels. Extracellular cAMP can be metabolized by ecto-phosphodiesterases to adenosine monophosphate (5′-AMP), and then by ecto-5′-nucleotidases to Ado (Conde et al., 2009). Interestingly, extracellular cAMP can modulate phenotype, function and differentiation of human monocytes through A_2A_ and A_2B_ Ado receptors (Sciaraffia et al., 2014). The contribution of extracellular cAMP to Ado production/Ado receptors activation has never been investigated in the CB, and cAMP released from the CB has never been quantified.

Intracellular cAMP can diffuse intercellularly through well-characterized gap junctions (Bennett et al., 1991; Bevans et al., 1998; Bevans and Harris, 1999). In the rat CB, connexin 43 (Cx43) gap junctions are found between type I cells and carotid nerve terminals, and mediate intercellular communications and transport of small molecules and ions (Abudara and Eyzaguirre, 1996; Eyzaguirre, 2007). Electrical coupling, gap junction formation and connexin expression are regulated by cAMP (Abudara and Eyzaguirre, 1998; Abudara et al., 1999, 2000; Eyzaguirre, 2007) and chronic hypoxic exposure (Chen et al., 2002a).

All together, these findings suggest different regulations of cAMP signaling in the CB, mediated not only by the enzymes directly involved in the synthesis and degradation of this signaling molecule, but also by a variety of effectors that can modulate its accumulation, and consequently, trigger changes in the CB activity.

### Current understanding of the role of cAMP-signaling pathway on the overall carotid body chemosensitivity

Observations from our laboratory show that cAMP levels are higher during normoxia than in hypoxic or hyperoxic superfusate in the whole CB from young and adult rats (Monteiro et al., 2011). These results support our view that cAMP-pathway may be involved in the maintenance of basal activity of the CB (translated in basal release of NTs or basal CSN electrical activity), suggesting a homeostatic and/or adaptative role for the cAMP-pathway in the rat CB. Although, inter-species differences may exist (Delpiano and Acker, 1984, 1991; Wang et al., 1989; Pérez-García et al., 1990; Cachero et al., 1996; Chen et al., 1997). Findings from experiments studying NTs systems using CB from rabbits vs rat and cat often differ. For example, dopaminergic influence on CB function is excitatory in the rabbit while it is inhibitory in CB from other mammalian species (Fidone et al., 1982; Vicario et al., 2000a), with higher DA secretion in rats, combined with lower cAMP accumulation (through D_2_ receptor activity).

Together with the other reported actions of cAMP signaling in the CB (Table 3), our results are consistent with the view that the cAMP-pathway is involved in the maintenance of basal CB excitability and thus the basal release of NTs and CSN sensory discharge frequency. The observed reduction in hypoxic sensitivity when cAMP signaling is targeted indicates that basal levels of cAMP act to prime the CB to subsequent hypoxic stimulation. This is likely achieved through the basal regulation of PKA and EPAC and possibility other, as yet unidentified downstream effectors. Given the synergy between hypoxia and hypercapnia, the basal type I cell [cAMP]_i_ may also confer the excitability to high CO_2_ or H^+^ although further evidence is required to confirm this. It would be of considerable interest and relevant to humans to determine how chronic hyperoxia and/or hypercapnia modifies cAMP signaling in the CB.

**Table 3:** **Effects mediated by cAMP signaling in the carotid body**.

**Effects mediated by cAMP**	**Preparation**	**Technique**	**Agents that modulate [cAMP]**	**References**
Increase of junctional conductance	Type I cells (young rats)	Dual-voltage clamping	dB-cAMP (1mM), 8-Br-cAMP (1 mM, 3 h)	Abudara and Eyzaguirre, 1998
Increase of the tyrosine hydroxylase gene expression elicited by hypoxia	Whole CB (adult rats)	Reverse-Transcriptase- polymerase chain reaction	FSK (0.01 mM, 3 h)	Chen et al., 1995
Activation of Cl^−^ currents	Type I cells (P10 rats)	Patch-clamp/whole-cell recording	cAMP (0.2 mM) 8-bromoadenosine-cAMP (2 mM)	Carpenter and Peers, 1997
Increase of Na^+^ and Ca^2+^ inward currents and capacitance	Type I cells (P5-12 rats)	Patch-clamp/whole-cell recording	dB-cAMP (0.2–1 mM) and FSK (0.01 mM) up to 15 days	Stea et al., 1995
Induction of Na^+^- channels and hypertrophy of type I cells	Type I cells (P5-12 rat)	Patch-clamp/whole-cell recording	Bt2-cAMP (1 mM, upto 14 days)	Stea et al., 1992
Potentiation of (30% O_2_)-evoked CA release and CSN discharge	Whole CB, CSN (adult rabbit)	CSN activity recording; CA release	FSK (0.01 mM, 10 min)	Wang et al., 1991a
Increase of DA release elicited by hypoxia (5% O_2_)	Whole CB (adult rabbit)	CA release	FSK (5–10μM), dB-cAMP (2mM) IBMX (0.5 mM), ISO (0.01–0.050 mM)	Pérez-García et al., 1990, 1991, 1993
Elevation of Ca^2+^- currents (mimic the effect of hypercapnia)	Type I cells (adult rabbit)	Whole-cell recording	8-Br-cAMP (0.5 mM, 10 min)	Summers et al., 2002
Increase of GAP-43 and neurofilament (NF68 and NF160 kD) expression and neurite outgrowth	Type I cells (P5-7 rat)	Double-label immunofluore scence	dB-cAMP (1mM), FSK (0.01 mM), up to 2 weeks	Jackson and Nurse, 1995

dB-cAMP or Bt2-cAMP, Dibutyryl-cAMP; 8-Br-cAMP, 8-Bromoadenosine-cAMP; FSK, Forskolin; P, Postnatal day; CA, catecholamines; CSN, Carotid Sinus Nerve; DA, Dopamine; ISO, Isoprostane; IBMX, Isobutyl-1-methylxanthine; GAP-43, Growth-Associated Protein 43; CB, carotid body.

### Novel techniques to study cAMP signaling in the carotid body

With tmACs restricted to membranes, and sAC, PDEs and cAMP effectors widely distributed within the cells, cAMP accumulation is spatially and temporally controlled, generating cAMP microdomains. Thus, a comprehensive study of cAMP signaling should complement the cAMP quantifications made in CB homogenates using RIA (Pérez-García et al., 1991; Wang et al., 1991a; Cachero et al., 1996) or EIA (Batuca et al., 2003; Conde et al., 2008; Nunes et al., 2010, 2013; Monteiro et al., 2011), which are themselves static and terminal assays. Measurements in intact type I cells using reporter protein constructs may allow for a more detailed quantification of cAMP in distinct subcellular compartments. Nowadays, a variety of FRET-based biosensors are available to visualize signaling dynamics in living cells (Sample et al., 2014). The literature is void of data obtained using life imaging techniques to manipulate cAMP in the CB. Our laboratory in collaboration with the laboratory of Dr. Jin Zhang, have been successful in using FRET-based reporters in CB type I cells to interrogate cAMP-signaling pathways (Nunes et al., 2013).

Image-based techniques can be used to understand interactions between cAMP and other mediators that raise [Ca^2+^]_i_ levels. Intensification of [Ca^2+^]_i_ signals have been linked with raised levels of angiotensin II, endothelin-1, cytokines, insulin and free radicals along with decreases in nitric oxide: levels of these substances are changed in disease states, associated with CB dysfunction (Chen et al., 2002b; Rey et al., 2006; Fung et al., 2007; Li et al., 2010; Schultz, 2011; Del Rio et al., 2012; Lam et al., 2012; Ribeiro et al., 2013). Intracellular Ca^2+^ levels can influence cAMP signaling directly through modulation of the activity of PDE and AC isoforms or indirectly through PKC activity, not only by allosteric regulation, but also by desensitization of GPCRs. In this sense, a compensatory cAMP mechanism may function to partially restore some sort of homeostatic control within the type I cell despite serious remodeling of the sensory transduction cascade in CB dysfunction.

As described above, our current view is that cAMP has an important role in the CB homeostasis, as suggested by the higher cAMP levels under normoxic conditions. Understanding how [Ca^2+^]_i_ and cAMP signaling is modified in conditions that lead to CB dysfunction, will be fundamental to understanding the role of these second messengers in the CB transduction/neurotransmission mechanism in health and disease.

## Is carotid body implicated in the effects of drugs that modify cAMP for therapeutic purposes?

**Figure 1:**
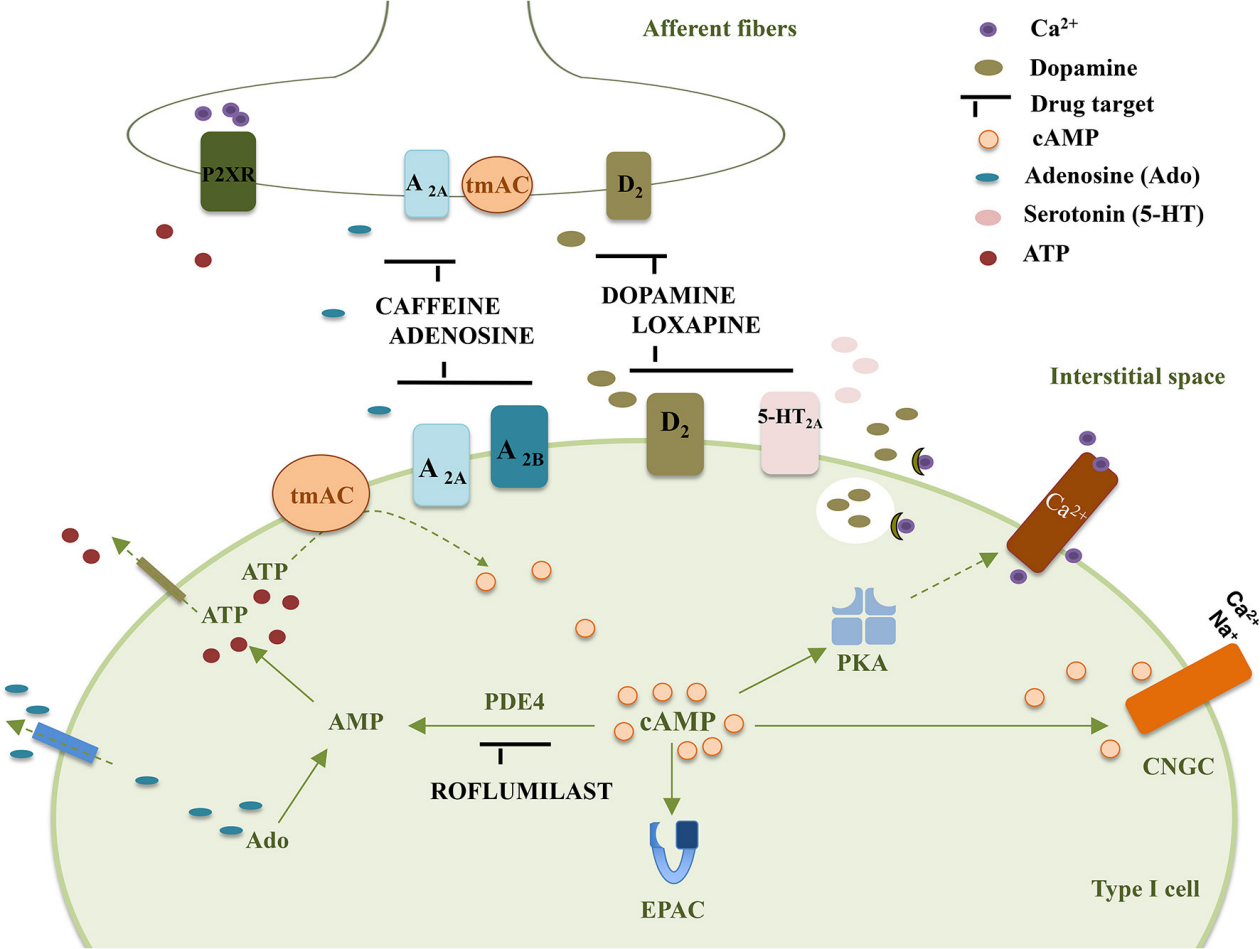
**Representation of some drug targets in type I cells and CSN endings that affect cAMP accumulation in the carotid body**. tmAC, transmembrane Adenylyl Cyclase; PKA, Protein Kinase A; EPAC, Exchange Protein Activated cAMP; D_2_, Dopamine receptor D_2_; A_2A_, Adenosine receptor A_2A_; A_2B_, Adenosine receptor A_2B_; 5-HT_2A_, serotonin receptor 5-HT_2A_; P2XR, ATP ionotropic P2X receptor; Ado, adenosine; PDE4, Phosphodiesterase 4; CNGC, Cyclic Nucleotide Gated channel.

The following section focuses on the CB mediated effects induced by currently clinical therapy that target cAMP-signaling. It is not aimed to extensively describe the putative drug effects that can be mediated by the CB but only to give insights that can stimulate future research in a field where the information is scarce. Figure 1 summarizes molecular targets in type I cell and CSN endings that may be affected by drugs-induced changes in cAMP accumulation.

*Exogenous DA* is extensively used in human to improve cardiac output and peripheral perfusion in patients with cardiogenic and septic shock. Several years after using DA, its inhibitory effects on ventilation in man were described and attributed to an effect on chemoreceptor reflexes (Welsh et al., 1978). Twenty years later, Van de Borne et al. (1998) showed that repeated use of DA impairs the ventilatory response to hypoxemia due to an inhibitory effect on CSN activity, which explains why when administered in low doses to conscious patients, DA reduces the discomfort caused by hypoxemia. Although its clinical use as vasopressor remains, both DA and NA have been used: DA decreases CB activity while NA does not appear to have an effect on CB activity (Zapata, 1975; Debaveye and Van den Berghe, 2004).

The impact of chronic use of *antipsychotics* (D_2_ antagonists) on peripheral chemoreflexes is unknown but beneficial effects of loxapine on agitation and breathing patterns during weaning from mechanic ventilation have been described (Sztrymf et al., 2010). These findings open doors to a promising field to explore CB manipulation to improve adaptation to mechanical ventilation.

Acute administration of *Ado* (full agonist of A_2A_ and A_2B_ receptors) is clinically useful to revert paroxysmal supraventricular tachycardia and causes dyspnea and chest discomfort mediated by CB activation (Watt et al., 1987; Reid et al., 1991). *Caffeine* is a non-selective Ado antagonist that has been used to prevent and treat apnea of prematurity due, primarily, to the blockade of inhibitory Ado A_1_ receptors in the CNS. Moreover, the effects of chronic coffee consumption have been extensively studied in the last years and it is now consensual that coffee, and probably caffeine, may reduce the risk of type 2 diabetes mellitus and hypertension, as well as other conditions associated with cardiovascular risk such as obesity and depression (O'Keefe et al., 2013). In fact, research in our laboratory have shown that chronic caffeine intake decreases circulating CAs, prevents diet-induced insulin resistance and hypertension (Conde et al., 2012b) and restores insulin sensitivity in aged rats (Guarino et al., 2013). Knowing that at the CB, caffeine blocks excitatory Ado A_2A_/A_2B_ receptors (Conde et al., 2006b) and that CB denervation prevents the development of insulin resistance and hypertension induced by hypercaloric diets (Ribeiro et al., 2013) the CB modulation by caffeine can improve conditions associated to sympathetic mediated CB hyperactivity (e.g., hypertension). Other effects of caffeine have been described in the CB e.g., mobilization of Ca^2+^_i_ stores in the CB cells by ryanodine receptor activation (Vicario et al., 2000b; Mokashi et al., 2001). However, they do not seem to be relevant in the clinical setting because their effects are achieved with toxic concentrations (Fredholm et al., 1999).

*Roflumilast*, an oral selective PDE4 inhibitor, was approved in 2011 by both the Food and Drug Administration (FDA) and the European Medicines Agency (EMA) for the treatment of severe chronic obstructive pulmonary disease (COPD), due to its anti-inflammatory and bronchodilator effects. No evidences of roflumilast effects on CB activity have been reported and those are difficult to address essentially because of their CNS effects and the variability of the PDE inhibitors efficacy in hypoxic conditions (Nunes et al., 2010). Curiously, the roflumilast efficacy to reduce the risk of COPD exacerbations has only been shown in patients that experience reduced dyspnea (Rennard et al., 2014). Since CB resection relieves dyspnea in COPD patients and improves FEV_1_ (Force Expiratory Volume) (Whipp and Ward, 1992) but exarcebates hypoxemia and hypercapnia and overall worsen the long term outcome (Stulbarg et al., 1989), the link between the mechanism of roflumilast action in COPD patients and CB activity merits further studies.

From the above evidence, one can conclude that the manipulation of cAMP signaling pathway is important to address O_2_/CO_2_ related diseases. However, manipulation of cAMP signaling may have consequences in the CB, that are clinically relevant and that have not yet been identified.

## Conclusions

The importance of cAMP to CB physiology has moved from a discarded player in the O_2_ chemotransduction to a central signaling molecule that is converged upon by multiple NTs/NMs, which collectively maintain an equilibrated CB activity. It remains to be seen whether modification of cAMP can improve patient outcomes in diseases associated with CB impairment or hyperactivity. We suggest that cAMP has an important role in the homeostasis of the CB since cAMP levels seem to be higher under normoxic conditions. Despite the increase in knowledge of CB physiology, the activity of tyrosine hydroxylase is still the hallmark of the CB and cAMP the classical second messenger of dopamine D_2_ receptor signaling.

Understanding how calcium and cAMP cooperate in dysfunction CB, will be fundamental to understand the role of these second messengers in the CB transduction mechanism. Additionally, systemic pharmacological manipulation of cAMP signaling can have clinically relevant consequences mediated by the CB. This may proven to be an exciting field of research that is still currently unexplored.

### Conflict of interest statement

The authors declare that the research was conducted in the absence of any commercial or financial relationships that could be construed as a potential conflict of interest.
